# Time-Dependent Exposures and the Fixed-Cohort Bias

**DOI:** 10.1289/ehp.1103885

**Published:** 2011-10-01

**Authors:** Adrian G. Barnett

**Affiliations:** Institute of Health and Biomedical Innovation, School of Public Health, Queensland University of Technology, Kelvin Grove, Queensland, Australia, E-mail: a.barnett@qut.edu.au

Hwang et al. (2011) showed an interesting association between air pollution and stillbirth. The authors examined births between 1 January 2001 and 31 December 2007 in Taiwan using a case–control design, with each of 9,325 stillbirths matched to 10 controls. They examined exposures from all three trimesters. For cases and controls born before September 2001, some exposures could have occurred in March–December 2000. Other pregnancies with exposures during this period could not be included in the study because the births occurred before January 2001, thus having the potential to bias the estimates of time-dependent exposures such as air pollution. We previously labeled this bias the “fixed-cohort bias,” but it applies equally to case–control designs using fixed dates of birth to recruit subjects (Strand et al. 2011).

For example, for pregnancies in their first month during June 2000, we can assume that some will result in stillbirth. Stillbirths often have relatively short gestations; therefore, some of these stillbirths would not be included in the cohort because they would have occurred before January 2001. In contrast, live births from this time could have made it into the cohort. In Table 1 of Hwang et al. (2011), mean gestation time was 26.9 weeks for the stillbirth subjects and 38.5 weeks for the control subjects. So for pregnancies in their first month in June 2000, the mean date of birth for stillbirths would be in December 2000 (outside the cohort), whereas the mean date of birth for live births would be in February 2001 (inside the cohort). This means that first trimester exposures during June 2000 may look remarkably protective, as the number of stillbirths would be very small. The bias for a study of air pollution would then depend on what exposure occurred in June 2000 and what the true association is. If it was a month with a particularly high level of air pollution, this would bias any true association between pollution and stillbirth towards the null. If there was no association between pollution and stillbirth, the bias would be toward a false finding of a protective effect.

The bias can also occur at the end of the cohort, with the longer pregnancies missed and the shorter pregnancies captured.

There is a simple way to avoid the bias: by excluding case and control subjects with estimated conception dates 20 weeks (shortest gestation) before the data collection started or 43 weeks before it ended (assuming a longest gestation time of 43 weeks). This ensures that the exposures examined during any gestation period could equally apply to cases and controls. The cost is a loss of sample size, which may widen any confidence intervals. I estimate that around 7% of pregnancies would need to be excluded by Hwang et al. (2011), but the benefit would be the removal of a potentially serious bias.
